# A Study on Instructional Humor: How Much Humor Is Used in Presentations?

**DOI:** 10.3390/bs12010007

**Published:** 2021-12-29

**Authors:** Vera Paola Shoda, Toshimasa Yamanaka

**Affiliations:** 1 Center for Computational Social Science (CCSS), Research Institute for Economics and Business Administration (RIEB), Kobe University, 2-1 Rokkodai, Nada, Kobe 657-8501, Japan; 2 Degree Programs in Systems and Information Engineering, Graduate School of Science and Technology, University of Tsukuba, 1-1-1 Tennodai, Tsukuba 305-8573, Japan; 3 Graduate School of Comprehensive Human Sciences, University of Tsukuba, Tsukuba 305-8577, Japan; tyam@geijutsu.tsukuba.ac.jp

**Keywords:** laughter, humor, presentations, instructional humor, quantitative, computational linguistics, frequency

## Abstract

Humor is applied in pedagogy to create a positive learning environment. Recent research focuses on the theories, effects, individual differences, and qualitative aspects of humor for instruction. However, there is a lack of studies focusing on quantitative features. Therefore, this research explored the quantitative characteristics of instructional humor in a naturalistic setting and applied techniques from natural language processing (NLP). This paper describes the results of two studies. The first study focused on instructional humor frequency and the placement of humor, while the linguistic features of instructional humor and non-instructional humor were compared in the second study. Two corpora were used in this research: TED Talks and user-submitted jokes from “stupidstuff.org” The results found that educators used humor 12.92 times for popular talks, while less popular talks only had 3.92 times. Humor is also more commonly placed during the first parts of the talk and lessens toward the end. There were also significant differences between the linguistic features of instructional and non-instructional humor in terms of readability scores and sentiment. These results provide a substantial update on quantitative instructional humor research and help educators understand how to use humor in the classroom in terms of quantitative and linguistic features.

## 1. Introduction

Educators face the challenge of creating a learning environment that is advantageous to student learning. Especially with the rapid changes and advances in society, new methodologies and tools for education are highly valuable. Humor is an essential tool used by educators to improve the learning environment of their students. The use of humor is a prevalent communication behavior in pedagogical settings. In 1983, Robinson argued that “What is learned with laughter is learned well.” [1] (p. 121). Likewise, much research provides evidence that humor positively affects student learning [2,3,4,5,6,7,8,9,10,11,12,13,14]. Therefore, research in instructional humor is significant to educators.

### 1.1. Definition and Theories of Humor

Researchers define humor in a variety of ways. According to Scheel [15], superiority, incongruity, and arousal relief are the most popular theories in humor research. Superiority theory, which has been prevalent since the time of Plato and Aristotle, explains that laughter is an effect of a feeling of superiority due to the depreciation of other people [16]. Incongruity theory argues that something is perceived as humorous when there is a contradiction or unexpected outcome [17]. In the arousal theory, Berlyne [18] said that “Humorous situations always contain factors that can be expected to raise arousal and other factors that can be expected to lower arousal or else keep it within moderate bounds.” [18] (p. 861). Another interesting theory in humor research is the anxiety theory, which states that laughter results from tension release [19].

### 1.2. Humor and Laughter

Laughter is more ancient than humor or speech [20] and is considered a social stimulus [21]. Similarly, laughter is also defined as a component of a universal language of basic emotions, which all people have in common and recognize [22,23]. According to Willman, “Laughter occurs when a total situation causes surprise, shock, or alarm, and at the same time induces an antagonistic attitude of playfulness or indifference.” [24] (p. 70). Several works in humor recognition have used laughter to indicate an instance of humor [25,26]. However, literature is divided on the relationship between humor and laughter. Some researchers argue that laughter is a physical manifestation of humor, while others believe the opposite. Laughter is a phonetic activity, while humor is treated as a cognitive concept [27]. On the contrary, humor is a psychological state characterized by the likelihood to laugh wherein contradictory ideas are held simultaneously [11,28]. Other research supporting the humor and laughter relationship has proved that laughter occurs in all the theories of humor, whether they be the superiority theory, incongruity theory, or the relief theory [29]. While we recognize that not all instances of laughter are effects of humor and not all humor can elicit laughter, in this paper, which follows research supporting the relationship between humor and laughter, we consider laughter as an indication for humor usage in the learning environment.

### 1.3. Theories of Instructional Humor

Since humor has several functions aside from being a tool used in education [30], in this paper, we will refer to humor used in an educational context as instructional humor. In the academic context, the usage of humor in the classroom has proved to have positive effects such as generating attention and arousing curiosity [31]. Prominent theories in instructional humor include the instructional humor processing theory (IHPT), which explains that the students need to perceive and solve the paradox in humor to ease their learning [32]. Another related research links the cognitive load theory [33] to humor application in STEM education [34]. According to Hu et al. [34], humor in STEM education should be integrated into the intrinsic cognitive load to be effective. The studies in instructional humor can be divided further into quantitative, qualitative, or individual differences; effects; and theories [30]. IHPT also states that, for instructional humor to have positive results, students must be motivated and comprehend the lesson content [13,32,35]. Application of the IHPT at different levels of education and learning environments has similar effects and conditions. For instance, at the higher-education level, students’ cognitive learning is predicted by the instructor’s related humor [36]. In online social networks, instructors’ humorous posts improved student engagement and instructor credibility [37,38]. Application of IHPT was also observed in a study in fifth- to tenth-grade students wherein teacher humor had to be related to the course to affect the learning experience positively [39]. Likewise, for adult learning environments in which students come from different nationalities and cultures, it is said that humor also increases the cultural competence and metalinguistic awareness of the students [40]. Regarding gender research, past research findings show that humor used by male instructors was more effective than that of female instructors [41].

### 1.4. Quantitative Instructional Humor

While research in instructional humor is abundant, there is a significant lack of works focusing on the quantitative aspects of humor [30]. Examples of research in quantitative instructional humor focus on instructional humor frequencies. For instance, Bryant et al. [42] found that professors utilized humor once every 15 min during a 50 min class session. In contrast, in similar research, Javidi and Long [43] found an average of 4.05 humorous messages. Likewise, Downs et al. [44] found that acclaimed professors used an average of 7.44 instances of humor per class. Other research includes that by Gorham and Christophel [13], who found only 1.37 humor attempts per class.

On the other hand, more recent research on instructional humor frequency focuses on self-reported measures. Moreover, frequency counts were obtained via survey forms or ratings rather than through actual counts of humor instances [45,46,47,48]. However, using self-reported measures yields lower rates of instructional humor [30]. There is a great need for studies that update our understanding of the field. While those studies provide extensive results, the learning environment and educational settings have changed drastically, mainly with the applications of technology such as e-learning [30]. Therefore, we conducted experiments on the instructional humor frequency in modern educational settings using a naturalistic approach. In addition, we also compared the linguistic features of instructional humor and non-instructional humor to provide educators with insights on how instructional humor differs from humor used for non-educational purposes.

### 1.5. Education Technology (EdTech) and E-Learning

Education technology (EdTech) is a growing field wherein information communication technology (ICT) supports learning and instruction [49]. Some examples of EdTech are e-learning [50], mobile learning [51], gamification [52], virtual reality and augmented reality platforms [53], and virtual meeting platforms [54]. One of the most discussed educational technology applications is e-learning, which uses the Internet to access educational materials outside traditional classrooms [52]. Some of the popular instructional content providers in e-learning include online course providers (Udacity [55], Course Hero [56], and Coursera [57]), language-learning website and mobile apps (Duolingo [58] and Rosetta Stone [59]), game-based learning platforms (Kahoot! [60] and Quizlet [61]), and online conference lecture streaming sites (TED Talks [62] and Talks at Google [63]). These providers heavily innovate the education field. Past literature revealed that these EdTech platforms impacted the learning environment with positive effects such as increased student performance [64], the lessened workload of teachers [65], and heightened student and teacher engagement [66]. E-learning gained popularity among educators and students for several reasons, such as functionality and affordability [67], social interaction [67], and collaborative learning [68].

### 1.6. Natural Language Processing (NLP)

The literature describes natural language processing (NLP) as using computational techniques to analyze texts at single or multiple levels of linguistic analysis, which aims to output human-like language processing in various tasks [69]. NLP is built on mathematical and linguistic foundations. NLP heavily draws from elementary probability theory and essential information theory in mathematics and parts of speech, morphology, semantics, and pragmatics in the linguistics field [70]. NLP techniques are applied in humanities, natural sciences, and social sciences research. NLP can help researchers in text data analysis by performing tasks such as assessing subjectivity, linguistic features, and classification. Examples of NLP techniques include sentiment analysis, which can classify text as negative, neutral, or positive [71]. Other methods include named-entity recognition (NER), wherein important nouns and pronouns are identified in a text [72], and sentence segmentation, which splits a large chunk of text into sentences [73].

### 1.7. Corpora: TED Talks and User-Submitted Jokes

Previous studies in quantitative instructional humor used corpora from lectures conducted in offline traditional classroom settings [42,43,44]. However, since we are interested in assessing instructional humor frequency in non-traditional classroom settings, this paper presents and discusses research findings from two studies using two corpora—TED Talks [62] and user-submitted jokes from stupidstuff.org (https://stupidstuff.org (accessed on 1 March 2021)) [74].

TED Talks are recordings of presentations done at TED conferences and related TED events. Talks vary in length, with most being about 20 min in length. Presenters in these talks come from various fields, and discussions range from science, design, technology, entertainment, and business, to global issues. The TED Talks corpus has been used in previous research on several topics such as resources for best practices in teaching [75], commenting behavior [76], academic listening exercises [77,78], and speech recognition [79]. In humor research, TED Talks are more used for building automatic humor recognition algorithms [25,26]. Although research on TED Talks is abundant, few have used it to describe the quantitative features of instructional humor. We chose the TED Talks corpus since the presentations follow a naturalistic setting wherein there were no restrictions on the humor usage of presenters, which is recommended by previous works [30].

Next, we also used user-submitted jokes from stupidstuff.org to represent non-instructional humor. We define non-instructional humor as sentences containing humor that was not initially intended for use in the classroom. Users can submit and publish their jokes on the website, and other users can also view and rate these jokes. The data on studpidstuff.org consist of jokes and their respective user ratings [74]. Research on humor using user-submitted jokes is also commonly used in building humor recognition systems and humor generation algorithms [80,81]. Thus, it is acceptable to use user-submitted jokes to represent non-instructional humor.

This paper presents and discusses research findings from two studies using these two corpora. In the first study, we observe the instructional humor frequency and the placement of humor using audience laughter in the TED Talks transcripts as a marker for humor instance. The second study compared the linguistic features of instructional humor and non-instructional humor. Natural language processing (NLP) techniques were applied in both studies, and all data analyses were conducted in the Python programming language.

Previous research has focused on self-reported methods [45,46,47,48] and offline settings [13,42,43,44] for calculating instructional humor frequency. This research is unique as we use corpora from online media (TED Talks and user-submitted jokes) and conducted the analysis using NLP techniques to better understand the context in a digital age. Past works have also only looked at traditional classroom settings. However, in this paper, we discuss findings in non-traditional classroom settings to account for the changing learning environment brought about by technological advances. These findings are relevant as they provide new insights into humor usage in online settings and update the literature on instructional humor frequency research. Our results also benefit teachers in incorporating humor in their lectures and engineers involved in NLP projects for humor recognition.

## 2. Materials and Methods

We conducted two studies in this research using computational linguistics and natural language processing (NLP) techniques. The field of computational linguistics is interdisciplinary and focuses on understanding both written and spoken language from a computational perspective [82]. Similarly, NLP is used to do text analysis using computerized approaches [83]. The research in this study was conducted using the programming language Python. For the first study, we focused on instructional humor frequency as found in presentations, while the second study compares the linguistic features of instructional and non-instructional humor. For this study, we set the alpha level for all statistical tests conducted in this research to 0.05.

### 2.1. Study 1: Instructional Humor Frequency in TED Talks

We used TED Talks as our corpus to examine the instructional humor frequency. Our study limited the talks to those conducted in the English language. We scraped to get the fifty most popular talks and the fifty least popular talks. To ensure that the least popular talks were not affected by the upload count date, we only considered talks that had been published at least one year earlier on the TED website. Likewise, only talks with a duration of 15 to 20 min were included in the dataset to make comparisons possible. We divided our corpus into the most popular and least popular talk datasets since previous works found significant differences in the instructional humor frequency usage based on the educator’s teaching experience and popularity among their students and peers [84]. Afterward, we extracted the transcript of the talks and created a command-separated values (CSV) file. We used the Pandas library [85] in Python to conduct the analysis.

While previous works had different methods to measure humor rates and locate humorous messages [13,42,43,44,45,46,47,48], in our study, we decided to locate the humor instances using the special markup "Laughter" found in the TED Talks transcripts. This markup occurs whenever the audience laughs during the presentations. We then examined these laughter occurrences using statistical tests. Furthermore, the placement of humor in the presentation timeline was analyzed. The results are explained in Section 3.

### 2.2. Study 2: Comparison of the Linguistic Features of Instructional and Non-Instructional Humor

In this experiment, we used two corpora: TED Talks and user-submitted jokes from stupidstuff.org. For the TED Talks, we extracted the transcripts of 2000 talks. After scraping the website, we created two CSV files for each corpus. We split the talks into sentences using the Stanza module (formerly the Stanford Core NLP) [86], then labeled sentences containing or immediately followed by the special markup "Laughter" in Python to get the sentences containing humor from the TED Talks transcripts. After data cleaning and processing, we were able to get 8906 humorous sentences from the TED Talks dataset. For stupidstuff.org, the dataset contained transcripts of 3200 user-submitted jokes. Finally, using NLP techniques and descriptive and inferential statistics, we looked at several linguistic features to compare the humorous sentences from the two corpora.

#### 2.2.1. Word Frequency, Bigrams, and Trigrams

Previous works identified that humor has a variety of functions, both positive and negative [87]. Therefore, it is vital to see whether there is a difference between the choice of words in instructional and non-instructional humor. To get the word frequency or the most frequent words appearing in our corpora and the most popular bigrams and trigrams of humorous sentences, we utilized Python’s open-source NLTK (Natural Language Toolkit) library [88].

#### 2.2.2. POS (Part of Speech)

Works in computational humor research found that humorous messages use personal nouns and proper nouns, such as when referring to human-related scenarios [89,90,91]. To see whether the humorous sentences in the instructional and non-instructional humor dataset follow this theory, we used an open-source library, TextBlob [92], in Python for POS tagging.

#### 2.2.3. Readability Score

We need to look at the readability score of the humorous sentences since the instructional humor processing theory (IHPT) emphasized that humor needs to be understandable and should not distract from the instructional message [32]. Likewise, the cognitive load theory (CLT) that focused on humor integrated into science, technology, engineering, and mathematics (STEM) education states that, if humor is not integrated into the lesson content, it will increase the students’ cognitive load and lower learning [33,34].

We used the Flesch reading ease and the Gunning Fog Index to calculate the readability of the humorous sentences. The Flesch reading ease scores range from 0 to 100, with 0 being extremely difficult and 100 being very easy to read [93]. On the other hand, the Gunning Fog Index rates text from 6 to 17, and each of these scores has an equivalent educational level that determines the text’s difficulty [94]. For example, a text with a Gunning Fog Index of 6 can be read by sixth-grade students, while a score of 17 can be read by college graduates [94]. We used the open-source library TextStat [95] and descriptive and inferential statistics in Python to conduct a readability score analysis.

#### 2.2.4. Sentiment Analysis

Research on the types of humor explains that the sentiment of the humorous messages can negatively or positively affect the listener [20,96]. For instance, humorous sentences having a negative feeling can lower students’ learning performance [20,96]. Thus, it is essential to conduct sentiment analysis on the instructional and non-instructional humorous sentences. We used the open-source library TextBlob [92] in Python to compute the polarity or sentiment of our dataset’s humorous sentences.

## 3. Results

### 3.1. Study 1: Instructional Humor Frequency

In this study, we first looked at how humor was placed throughout the presentation timeline. Then, we looked at the speakers’ frequency of humor usage and compared the results for popular and unpopular talks using the TED Talks dataset.

#### 3.1.1. Placement of Humor in the Presentation Timeline

**Figure 1:**
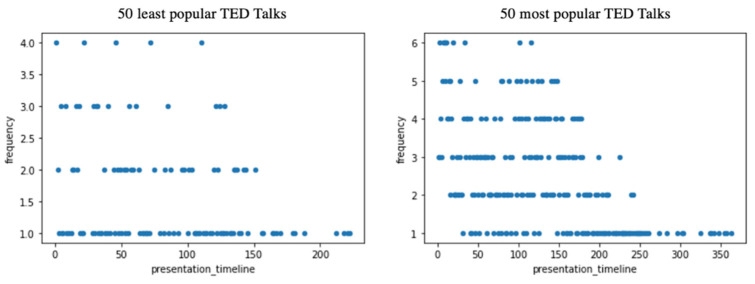
The frequency of audience laughter in the top 50 least popular (**left**) and top 50 most popular (**right**) TED Talks. The *x*-axis shows the presentation timeline or the *n*th position of the sentence when audience laughter occurred during the presentation. Frequency counts for each sentence’s *n*th position are shown in the *y*-axis.

The placement of humor in the presentation timeline was calculated using the sentence position when audience laughter occurred and its frequency (see Figure 1). We observed a maximum of six occurrences and four occurrences for the same sentence position in the presentation timeline for the most popular and least popular talks, respectively. Although popular talks had more observed laughter instances than less popular talks, the placement of humor in the presentation timeline seemed similar for both datasets. As shown in Figure 1, humor was more commonly observed during the first part of the talk and gradually lessened toward the end.

#### 3.1.2. Humor Frequency in Popular and Unpopular TED Talks

Popular talks incorporated humor an average of 12.92 times per 15 to 20 min, while unpopular talks only used humor for an average of 3.92 times. A Welch’s t-test on the humor frequency usage of popular and unpopular talks revealed that the difference was statistically significant between the two datasets (*p* < 0.001).

Instructional humor frequency usage in popular talks tended to vary more (M = 12.62, SD = 12.65) than in unpopular talks (M = 3.92, SD = 5.23). For instance, we found that the highest humor frequency for popular talks was 69 times while the lowest was 0 times. Out of 50 talks, 2 had zero humor usage for popular talks. On the other hand, 13 talks showed no humor usage for unpopular talks. Table 1 describes the statistics for the humor frequency in popular and unpopular talks.

### 3.2. Study 2: Linguistic Features of Instructional and Non-Instructional Humor

The results from NLP techniques such as calculation of word frequencies, *n*-grams, POS tagging, readability scores, and sentiment analysis applied to TED Talks and stupidstuff.org corpora are described in this section.

#### 3.2.1. Word Frequencies, Bigrams, and Trigrams

**Figure 2:**
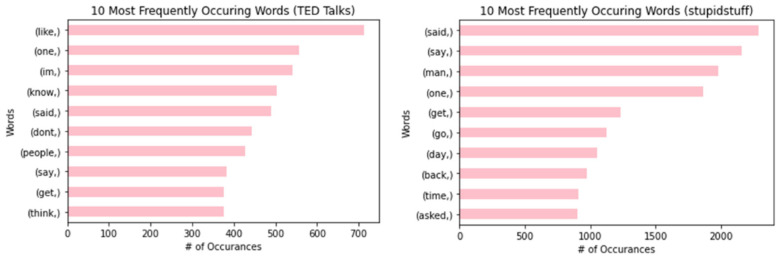
The 10 most frequently occurring words for humorous sentences in TED Talks (**left**) and user-submitted jokes from stupidstuff.org (**right**).

The top 10 most frequently used words for the TED Talks and stupidstuff.org datasets are described in Figure 2. The most common word for humorous sentences in TED Talks was “like”, and it was “said” for studpidstuff.org. The two datasets share some similarities in word frequencies. For example, the words “one”, “said”, “say”, and “get” both appeared in the top 10 most frequently occurring words for both datasets.

**Figure 3:**
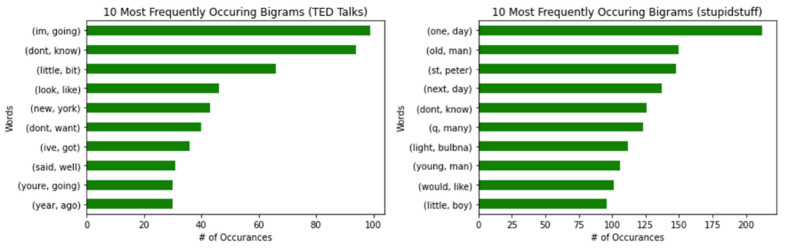
The 10 most frequently occurring bigrams for humorous sentences in TED Talks (**left**) and user-submitted jokes from stupidstuff.org (**right**).

Next, we looked at the most frequently used bigrams or two-word combinations. In Figure 3, we see that “I’m, going” and “one, day” were the most common bigrams for TED Talks and Stupidstuff.org datasets, respectively. Notably, the bigram “don’t, know” appeared in both datasets.

**Figure 4:**
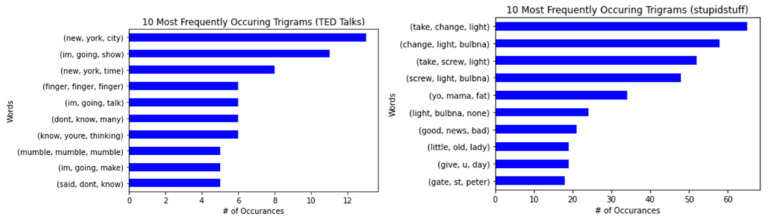
The 10 most frequently occurring trigrams for humorous sentences in TED Talks (**left**) and user-submitted jokes from stupidstuff.org (**right**).

Finally, the research looked at the most common trigrams (see Figure 4). “New, York, City” was the most frequently used trigram in the TED Talks dataset, and it was “take, change, light” for the stupidstuff.org dataset. We found no similarities between the two datasets regarding the most frequently used trigrams.

#### 3.2.2. Part of Speech (POS)

**Figure 5:**
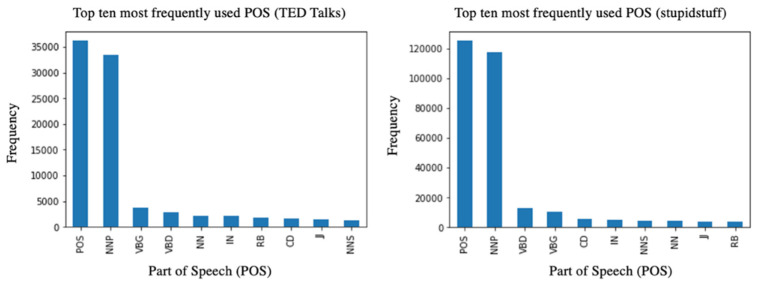
The 10 most frequently occurring POS for humorous sentences in TED Talks (**left**) and jokes from stupidstuff.org (**right**). Legend on POS tag definitions: POS (possessive ending), NNP (proper noun, singular), VBG (verb, gerund/present participle), VBD (verb, past tense), NN (noun, singular), IN (preposition/subordinating conjunction), RB (adverb), CD (cardinal digit), JJ (adjective), NNS (proper noun, plural).

We observed a high usage of possessive ending (POS) and proper nouns in singular form (NNP) for both datasets. This result supports previous works that state that humorous messages tend to use possessive forms and proper nouns [88,89,90]. Figure 5 below contains information on the two datasets’ top 10 most frequently used POS. Strikingly, the top 10 commonly used POS were similar for the two datasets. For example, the POS verbs in gerund or present participle form and verbs in the past tense form were frequently observed.

#### 3.2.3. Readability Score

Using the Flesch reading ease score, results showed that humorous sentences for both datasets tended to have scores from 60 to 100, with the peak at 80 and an average of 72–74. The results mean that the humorous sentences range from reasonably difficult to very easy to read. Humorous sentences from the TED Talks dataset (M = 73.89, SD = 17.32) were slightly easier to read and had minor variance compared to those of user-submitted jokes from stupidstuff.org (M = 72.45, SD = 18.88). Table 2 summarizes the results of the analysis. Welch’s t-test on the readability scores using the Flesch reading ease shows that they were statistically significant (*p* < 0.001).

**Figure 6:**
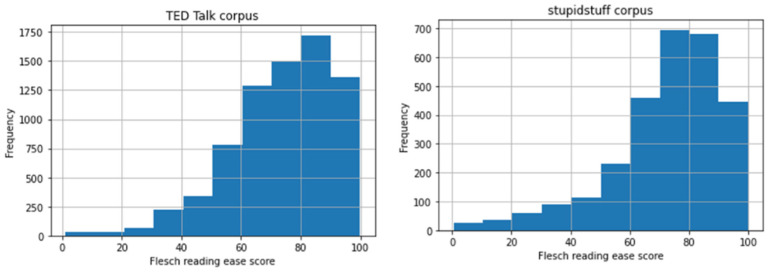
Histogram of readability scores for humorous sentences in TED Talks (**left**) and jokes from stupidstuff.org (**right**) using the Flesch reading ease method.

The number of samples used in this analysis removed outliers or talks that had readability scores out of the range of the scores determined by the Flesch reading ease score (see Figure 6). This result is favorable since it is recommended that instructional humor should be easy to understand and not increase the students’ cognitive load [32,33,34].

We also looked at the Gunning Fog Index for both datasets to further assess the readability of the humorous sentences. We removed outliers or talks that had readability scores out of range of the scores determined by the Gunning Fog Index in this analysis. Table 3 describes the results of the statistical tests. A Welch’s t-test on the readability scores using the Gunning Fog Index revealed that the difference was statistically significant (*p* < 0.001).

**Figure 7:**
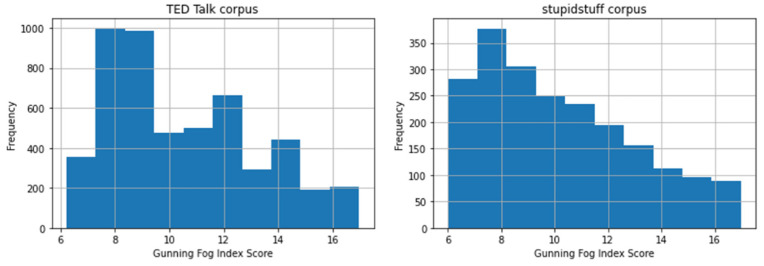
Histogram of readability scores for humorous sentences in TED Talks (**left**) and jokes from stupidstuff.org (**right**) using the Gunning Fog Index method. The score ranges from 6 (can be understood by sixth-grade students) to 17 (comprehensible to college graduate students).

Figure 7 describes the detailed results of the Gunning Fog Index assessment. The stupidstuff.org dataset (M = 10.19, SD = 2.82) tended to have slightly more variation in scores than the TED Talks dataset (M = 10.55, SD = 2.65). Nevertheless, both datasets returned scores with an average of 10, meaning lower grade levels can comprehend the sentences containing humor.

#### 3.2.4. Sentiment Analysis

**Figure 8:**
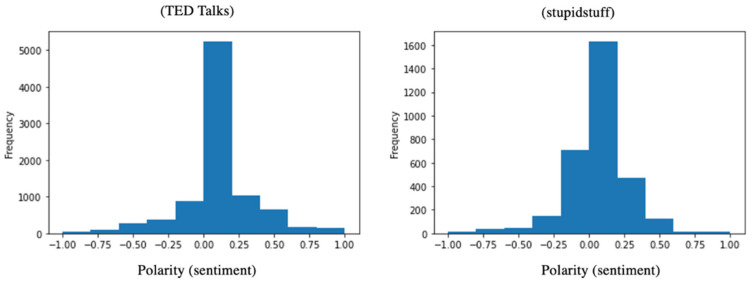
Histogram of sentiment for humorous sentences in TED Talks (**left**) and jokes from stupidstuff.org (**right**).

Figure 8 shows the histogram of the polarity of humorous sentences for the two datasets. We observed that humorous sentences for both datasets returned a neutral sentiment with an average of 0.07 and 0.06 polarity for TED Talks and stupidstuff.org, respectively. Negative scores imply a negative emotion, while positive scores indicate positive feelings, and near-zero scores usually express a neutral sentiment.

A Welch’s *t*-test on the sentiment scores of the two datasets revealed that the difference was statistically significant (*p* = 0.01). Table 4 summarizes the results of the statistical tests on the sentiment scores. The observations from the sentiment analysis of sentences containing humor in TED Talks (M = 0.07, SD = 0.27) and user-submitted jokes from stupidstuff.org (M = 0.06, SD = 0.21) showed that the two were very similar.

## 4. Discussion

This study aimed to investigate the quantitative and linguistic features of instructional humor in the EdTech learning environment and provide an update to quantitative instructional humor research by using techniques in NLP and statistical tests on presentations from TED Talks and user-submitted jokes on stupidstuff.org.

The results from our investigation on the frequency of instructional humor in TED Talks showed that educators’ incorporation of humor in their lectures had increased significantly (M = 12.62) as compared to the results from previous research dating back to three decades ago [24,25,26]. While earlier research findings in traditional classroom settings found humor usage once every 15 min [42], our research findings revealed humor usage once every 1.58 min. These findings show that humor usage by educators is significantly more frequent in non-traditional classroom settings. Learning environments in TED Talks incorporate more humor than traditional classroom settings do. The popularity of TED Talks among online viewers also follows past research that higher frequencies of humor applications receive higher satisfaction and popularity ratings from students [84]. Therefore, whether in traditional or non-traditional classroom settings, high frequencies of humor are associated with high popularity among audiences. Although, in this study, we were not able to identify what might cause the high humor frequency, this increase in humor usage in EdTech learning environments can be another point of interest for researchers to study.

On another note, we observed that the humor usage of more popular educators (M = 12.62) was significantly higher than that of less popular educators (M = 3.92). This observation is in line with previous research, wherein there is a correlation between the amount of usage of humor and the popularity, credibility, and experience of teachers [83]. Likewise, our results show that the humor used in TED Talks followed humor’s positive effects as stated in the instructional humor processing theory (IHPT) [32] since audiences express their positive experience through frequent laughter. This study confirms that despite changes in the learning environment such as those brought about by technology, the theory that more experienced, popular, and credible teachers tend to use humor more in their lectures [30] still holds.

Another important factor is that we looked at the placement of humor in the presentation timeline. This study is novel as far as we know, as previous studies only focused on the frequency of humor usage and not where humor was placed during the lectures. The results showed that humor is more commonly set during the start or first parts of the presentation rather than in the middle or end. These findings support the findings of previous works in which humor was used to seek attention [31]. Likewise, these results can also be linked to prior theories in instructional humor, wherein placing humor at the start of the lectures is more beneficial to student learning. To give a specific example, as suggested by Sweller in the cognitive load theory (CLT), humor should not add additional cognitive load for it to have positive effects [33]. Since the start of the lectures has the lowest cognitive load, it is understandable that the educators prefer early placement of humor in their classes. Similarly, as stated in the IHPT, since students’ cognitive load is lesser, students can quickly solve the contradiction of the humor, hence, increasing the positive effects of humor in their academic performance [32].

On the other hand, our comparison between the linguistic features of instructional humor and non-instructional humor provided several intriguing results. First, in terms of word frequencies, there seemed to be no difference between the word usage of humor used for education and that used for other purposes. Our findings support past studies wherein some words were more commonly used in humorous contexts, such as when referencing human-related scenarios [89]. In our results, words corresponding to human-centered scenarios such as “I’m”, “people”, and “man” are common in non-instructional and instructional humor cases. Similarly, previous studies suggest humorous sentences use more possessive pronouns and nouns [88,89,90]. The results from POS tagging are also in line with past literature since POS (possessive ending) and NNP (proper noun, singular) were the most frequently occurring POS for instructional and non-instructional humorous sentences. Our research further supports describing humorous sentences as human-centric and focusing on personal opinions, as observed in previous studies [88,89,90]. These results are also beneficial to the field of using NLP for building systems for humor recognition for machines since we can devise algorithms that take the linguistic components of sentences as features to recognize the humor in sentences automatically. Furthermore, the methodology of past works using words and pronouns as features for automatic humor recognition in machines [25,26,80,81] is also supported and validated through our research results.

When we looked at the readability scores of instructional and non-instructional humor sentences, we observed a slight difference in scores when using different methods for calculating the readability. Using the Flesch–Kincaid reading ease method [93], instructional humor had a higher mean, making it easier to understand. However, when using the Gunning Fog Index [94], non-instructional humor was much easier to comprehend. In this research, we only used two methods for calculating the readability score. Therefore, other researchers might apply other readability scoring methods to obtain comparable results.

Lastly, we expected to see more negative sentiment for non-instructional humor and more positive emotion for instructional humor in terms of sentiment analysis. However, both returned scores leaned more toward a neutral view. We expected a more positive sentiment for instructional humor since previous research suggests that humor used for education should contain positivity rather than negative feelings to create a positive learning environment [30]. Likewise, we expected to see more negative sentiment in non-instructional humor since past humor research studies showed that humor uses negative words, adult slang, and swear words [89]. Perhaps, the methodology we used for calculating the sentiment of the humorous sentences might not have been accurate enough, leading to neutrality. Since humor contains incongruity and ambiguity [17,32], the algorithm we used might not have detected the sentiment correctly. In future research, better algorithms and methods for sentiment analysis are recommended for getting more accurate results.

### 4.1. Implications of the Current Study

The current study provides teachers with insight into incorporating humor in their lectures in terms of quantitative features. The results also give a positive light to engineers in humor recognition research as even humor for different purposes tends to have the same linguistic features. The findings also update instructional humor frequency research, where the prominent studies date from 30 years ago with insights on the case of instructional humor frequency in non-traditional classroom settings.

### 4.2. Limitations and Future Research

The current study has several limitations. First, although we were able to find the results to be statistically significant, the number of samples we used to compute the instructional humor frequency and placement of humor in the presentation timeline was limited to a small sample size for both popular (N = 50) and unpopular talks (N = 50). Second, the method for calculating humor rates was highly dependent on the transcriptions of the presentations using the special markup “Laughter” since we did not conduct any test to validate whether “Laughter” instances accounted for humor usage. For instance, the audience’s laughter might have been caused by other factors and not by the actual humor usage of the presenter, but in this study, we assumed that the presenter incorporated humor when the audience in the talk laughed. Third, the presentation timeline in this study was expressed with the sentence position rather than the actual time. The reason for this was that the presentations had varying lengths (15 to 20 min), and we thought that occurrences would be better described by sentence position rather than time to help teachers plan their lectures using the sentence position or word count rather than time. In our future research, we will take time as the *x*-axis for the presentation timeline and study longer presentations (more than 20 min) and see whether there are any changes in the results. Fourth, the algorithms in this study for studying the linguistic features such as word frequencies, POS tagging, readability scores, and sentiment analysis of instructional and non-instructional humor were limited to those we used. In future research, we can try incorporating different algorithms for each analysis and see if there is a difference in the results. Fifth, we were only able to assess the effects of humor frequency regarding the popularity of the TED Talks and not determine the audience’s comprehension. In future studies, it would be interesting to see if high frequencies of humor in TED Talks positively or negatively affect the audiences’ learning performance. Sixth, we used non-traditional classroom settings in this study to observe instructional humor frequencies since we wanted to see how humor is used in the digital context. However, further research using offline lectures can also be of potential interest for more direct comparisons on how humor frequencies changed in traditional classroom settings. Lastly, we only used Welch’s t-test for statistical tests to confirm whether our dataset’s difference was significant. We can use more statistical tests to improve our findings in future research.

## 5. Conclusions

Overall, this study updates instructional humor frequency research by assessing humor usage in non-traditional classroom settings. We observed that educators’ use of humor increased significantly compared to research results 30 years ago. Humor usage in non-traditional classroom settings such as TED Talks is more frequent. On the other hand, this research finding supports previous theories stating that the more experienced, credible, and popular educators are, the higher their usage of humor. Whether in learning environments supported by technology or not, humor positively affects the presentations’ popularity. In addition, results showed that humor is more commonly placed at the start of presentations. This study also showed comparisons on the linguistic features between humor used for educational purposes and those used for other purposes. Whether for instructional or non-instructional purposes, humorous sentences follow similar linguistic features. Our findings are beneficial to teachers in incorporating humor in their lectures and to engineers involved in NLP projects for humor recognition.

## Figures and Tables

**Table 1:** Summary of statistics for the humor frequency of TED Talks.

	N	Minimum	Maximum	M	SD
Popular talks	50.0	0.00	69.00	12.62	12.65
Unpopular talks	50.0	0.00	30.00	3.92	5.23

**Table 2:** Summary of statistics for the readability scores using the Flesch reading ease score.

	N	Minimum	Maximum	M	SD
TED Talks	7348.00	1.09	99.91	73.89	17.32
User-submitted jokes	2839.00	0.43	99.94	72.45	18.88

**Table 3:** Summary of statistics for readability scores using the Gunning Fog Index.

	N	Minimum	Maximum	M	SD
TED Talks	5116.00	6.22	16.96	10.55	2.65
User-submitted jokes	2094.00	6.01	16.98	10.19	2.82

**Table 4:** Summary of statistics for sentiment analysis.

	N	Minimum	Maximum	M	SD
TED Talks	8906.00	−1.00	1.00	0.07	0.27
User-submitted jokes	3200.00	−1.00	1.00	0.06	0.21

## Data Availability

The data presented in this study are openly available on Kaggle at https://kaggle.com/vpshoda/instructional-humor-research (accessed on 23 November 2021).
